# Post-Operative Complications and Risk Predictors Related to the Avulsion of Lower Impacted Third Molars

**DOI:** 10.3390/medicina59030534

**Published:** 2023-03-09

**Authors:** Andrea Blasi, Alessandro Cuozzo, Renata Marcacci, Gaetano Isola, Vincenzo Iorio-Siciliano, Luca Ramaglia

**Affiliations:** 1 Department of Periodontology, University of Naples Federico II, Via Pansini 5, 80131 Naples, Italy; andreablasi79@gmail.com (A.B.); alessandro.cuozzo90@gmail.com (A.C.); enzois@libero.it (V.I.-S.); luca.ramaglia@unina.it (L.R.); 2 Department of Oral Surgery, University of Naples Federico II, Via Pansini 5, 80131 Naples, Italy; renata.marcacci@yahoo.it; 3 Department of General Surgery and Surgical-Medical Specialties, School of Dentistry, University of Catania, 95124 Catania, Italy

**Keywords:** impacted tooth, third molar, tooth extraction, oral surgery complications, inferior alveolar nerve injury

## Abstract

*Background and Objectives:* This prospective cohort study aimed to evaluate the onset and severity of pain and other complications following lower impacted third molar extraction and to identify potential risk predictors. *Materials and Methods:* Twenty-five patients were treated with at least one lower impacted third molar extraction. The primary outcome was the onset of post-operative pain, evaluated at 6 h, 12 h, 24 h, 48 h, 72 h, and 7 days. The secondary outcomes (trismus, edema, alveolitis, dehiscence, neuralgic injury, and suppuration) were recorded at 3, 7 and 21 days after oral surgery. A correlation analysis was performed to identify potential associations between patient- and tooth-related factors and VAS (Visual Analogue Scale) scale. When a statistically significant correlation was identified, a regression analysis was performed. *Results:* Most of the patients were female (84%) with a mean age of 25 ± 3 years; the reason for oral surgery was dysodontiasis in 60% of cases, while the most frequent Pell and Gregory class was BII (36%). The VAS scale showed the onset of mild pain at 6 h (44%), 12 h (48%), 24 h (68%) and 48 (68%) after surgery. Trismus, edema, and alveolitis were observed at 3-day (20%, 64% and 12%, respectively) and at 7-day (16%, 12% and 4%, respectively) follow-up. Neuralgic injury was reported in one case (4%). The linear regression analysis showed a statistically significant association (*p* < 0.05) between the duration of oral surgery and VAS scores at 6 and 12 h. Finally, the binary logistic regression identified systemic disease, Pell and Gregory classification, duration of oral surgery, VAS at 6 and 12 h, trismus, and edema at 3 and 7 days as predictive factors of post-operative complications. *Conclusions:* Within their limits, the results of this study suggest that the onset of post-operative complications increases in proportion to the duration of the surgical procedure.

## 1. Introduction

Third molars are the most commonly impacted teeth, with a frequency of up to 75% in the young adult population [1]. The reason is related to human evolution, as there has been a development of the neurocranium to the detriment of the splanchnocranium, whose dimensions have remained almost unchanged and consequently made the third molars redundant [2]. Therefore, the lack of space has meant that the eruption of third molars occurs very frequently in anomalous positions, often determining clinical symptoms such as pain, otalgia, odynophagia, and dysphagia [3]. Consequently, surgical avulsion represents one of the most common procedures in oral and maxillofacial surgery [1,4].

Several indications for impacted third molar (IMTM) extraction are described in the literature, including orthodontic and periodontal reasons, caries, pain, infections, association with cysts or tumors, damage to the neighboring teeth, and proximity to mandibular fracture lines or orthognathic surgical sites [5,6].

Pre-surgical planning is essential in order to perform the extraction safely, reducing discomfort and complications. Winter and Pell and Gregory classifications [6,7] are the most-used methods to evaluate the extractive pattern of IMTMs, giving information on the angulation and the position in the mandibular ramus, respectively. According to Bui et al. [8], the mesio-angular position is associated with a higher risk of post-operative complications. However, these classifications are based on the orthopantomographic aspect only, which is not enough to define the difficulty level of the surgery [9,10]. For this reason, many authors [6,11,12] reported classification systems based on cone-beam computed tomography (CBCT) analysis, offering more detailed information on radicular morphology and on the relationship with the inferior alveolar nerve (IAN) [13].

Bone density, age, sex, previous gnathological pathologies, and surgeon experience are other pre-operatory variables that can influence the difficulty of the intervention, although there is not a single classification that includes all these necessary parameters [9,10].

The main objective of IMTM surgery is avulsion with minimal intra- and post-operative complications. After careful pre-operative planning, access to the third molar should be performed from the buccal side by incision of a mucoperiosteal flap, and it is preferable to have an accurate odontomy rather than osteotomy, removing undercuts and safeguarding the alveolar bone [14]. In the case of multi-rooted teeth, it could be convenient to separate them using either a diamond bur mounted on a straight handpiece or piezo-surgical instruments [15].

Subsequently, after having achieved adequate mobility, the tooth can be extracted, removing any periapical lesion and smoothing the sharp bone edges. Finally, the suture must be performed without tension in order to give stability to the flap and the clot [16].

Moreover, Barone et al. [17] reported other techniques, including cryotherapy, piezo-surgery, and the application of platelet concentrates in the surgical site, in order to minimize early and post-operative complications [17].

Post-intervention pharmacological therapy is also described in the literature to reduce the risk of infection and to inhibit the release of the inflammatory mediators responsible for the acute response. The most common antibiotic therapy involves the use of amoxicillin: 500 mg every 8 h (or three times a day) after surgery for a total of 5 days [18].

While some authors concluded that antibiotic prophylaxis does not seem to contribute to better wound healing, less pain, and greater mouth opening after surgery [19,20,21], a recent systematic review reports that the most common protocol (i.e., amoxicillin and clavulanate per os) seems to guarantee high predictability and safety [22]. Therefore, while the routine use of antibiotics for IMTM remains debated, it is very common in dental practices.

Non-steroidal anti-inflammatory drugs (NSAIDs) and corticosteroids are instead indicated to prevent and/or reduce post-surgery discomfort such as pain and swelling [23]. Among these, the most used are ketoprofen, ibuprofen, paracetamol, and prednisone, used with different dosages depending on the patient [24].

Approximately about 65% of IMTM interventions are performed with minimal intra- and post-operative discomforts (pain, edema), and less commonly, they incur major complications (post-operative bleeding, trismus, bone fractures, and paresthesia) [13]. Mantovani et al. [15] reported a high incidence of alveolar osteitis, dehiscence, and suppuration in the immediate post-operative period [15]. The onset of these complications could have a substantial impact on the patient’s quality of life, thus making a correct surgical and preventive approach necessary [25].

Currently, there are many studies in the literature that evaluate the onset of complications following IMTM avulsion, although few of these focus on the analysis of possible predictive risk factors [26].

Others, while reporting the association with pre-operative risk factors, did not analyze patients’ perceptions of quality of life after intervention [24]. On the other hand, many studies identified only individual variables, reporting descriptive assessments and making the interpretation difficult because of a lack of data [25].

Therefore, the aim of this study was to evaluate the onset and severity of pain and other complications following IMTM surgery and to identify potential risk predictors.

## 2. Materials and Methods

The study was designed as a prospective cohort study. Patients who needed the avulsion of at least one impacted lower third molar from October 2019 to October 2021 were selected at the Department of Oral Surgery—University of Naples Federico II.

### 2.1. Eligibility Criteria

The patients were selected based on the following inclusion criteria:

- Male and female;
- Age ≥ 18 years;
- Non-smoking patients;
- Complete root formation of lower IMTMs based on computed tomography scan (CT scan);
- Followed up to 3-, 7- and 21-day post-intervention.

The exclusion criteria were:

- Pregnant or breastfeeding;
- Alcohol or drug abuse;
- Upper third molars;
- Presence of suspected neoplastic lesion close to the impacted tooth (based on CT scan);
- Presence of relevant medical history contraindicating surgical therapy;
- Patients who have not undergone periodical follow-up.

### 2.2. Outcome Measures

**Figure 1:**
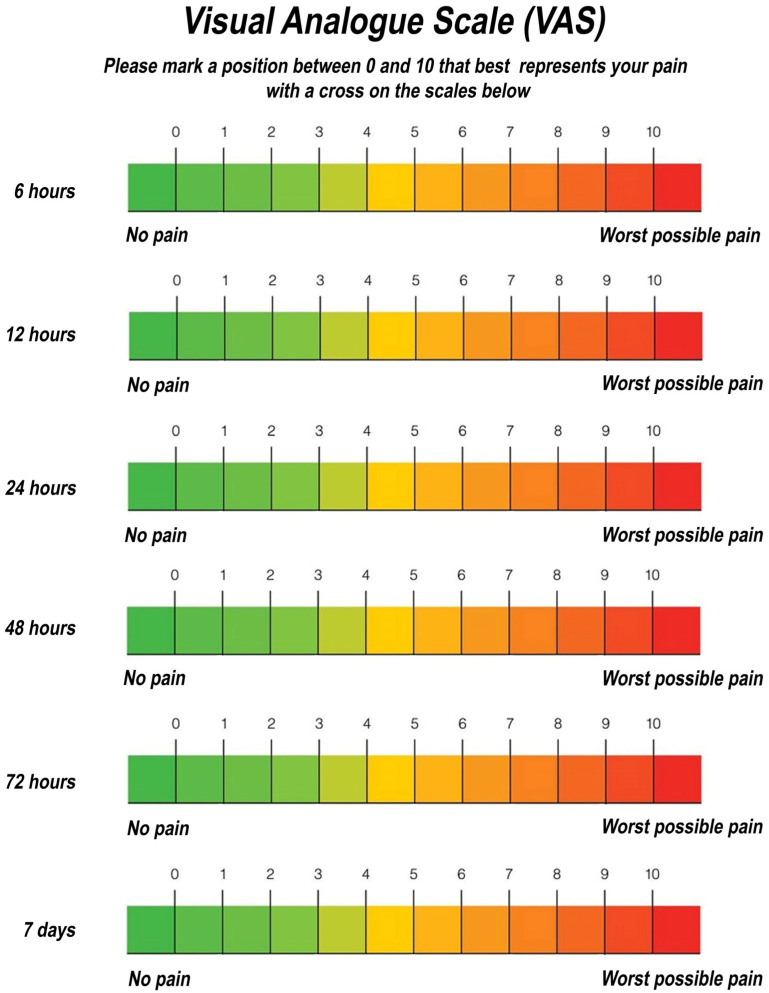
VAS scale.

The primary outcome was the onset post-operative pain, evaluated with a Visual Analogue Scale (VAS) at 6 h, 12 h, 24 h, 48 h, 72 h and 7 days (Figure 1). The VAS scores were recorded as follows:

- 0–1: absence of pain;
- 2–4: mild pain;
- 5–7: moderate pain;
- 8–9: severe pain;
- 10: extremely severe pain.

The secondary outcomes were the presence of trismus, edema, and other post-operative complications (alveolitis, dehiscence, neuralgic injury, and suppuration), all evaluated during periodical follow-up.

**Figure 2:**
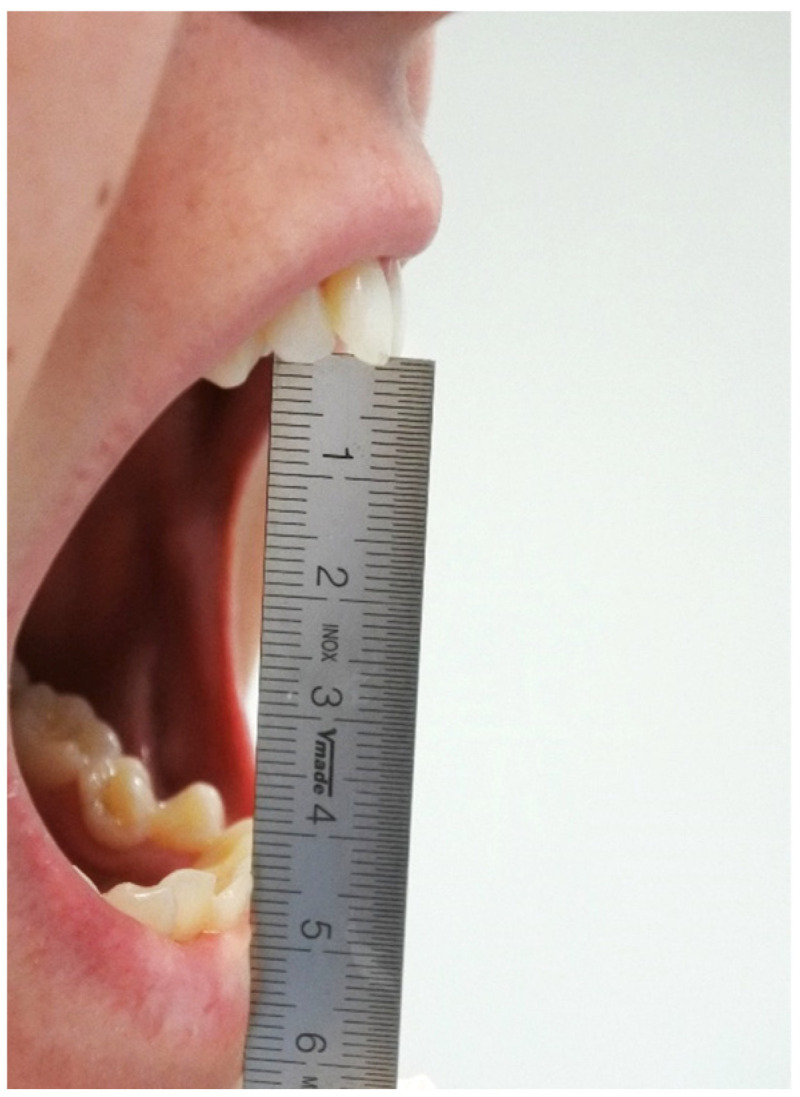
Measurement of the interincisal distance for trismus evaluation.

Trismus was assessed by measuring the difference between the interincisal distance pre-operatively and post-operatively at 3, 7, and 21 days (Figure 2). The measurements were performed using a millimeter ruler from the incisal edge of the maxillary central incisors to the incisal edge of the mandibular central incisors at the midline when the mouth was open as wide as possible (roughly 40–55 mm) [27].

**Figure 3:**
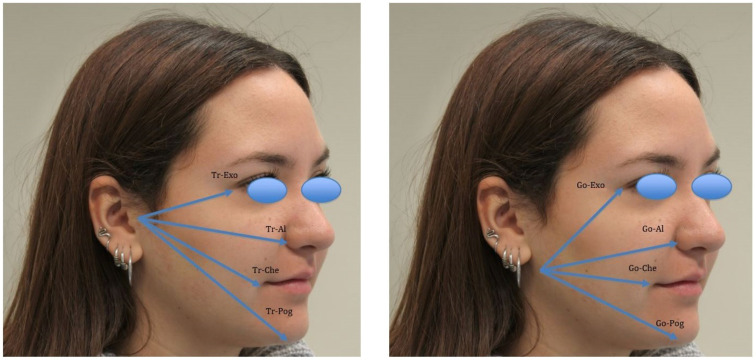
Facial and gnathological measurements for edema evaluation.

Post-operative edema was evaluated by analysing the differences between pre- and post-operative values (at 3, 7 and 21 days) of specific gnathological and facial measurements [28,29], performed with facial arches and millimeter rulers (Figure 3). The measurements were as follows:

- Trago to nasal border (Tr-Al);
- Trago to anatomic pogonion (TR-Pog);
- Trago to eye’s external corner (Tr-Exo);
- Trago to labial commissure (Tr-Che);
- Anatomic gonion to anatomic pogonion (Go-Pog);
- Anatomic gonion to eye’s external corner (Go-Exo);
- Anatomic gonion to nasal border (Go-Al);
- Anatomic gonion to labial commissure (Go-Che).

**Figure 4:**
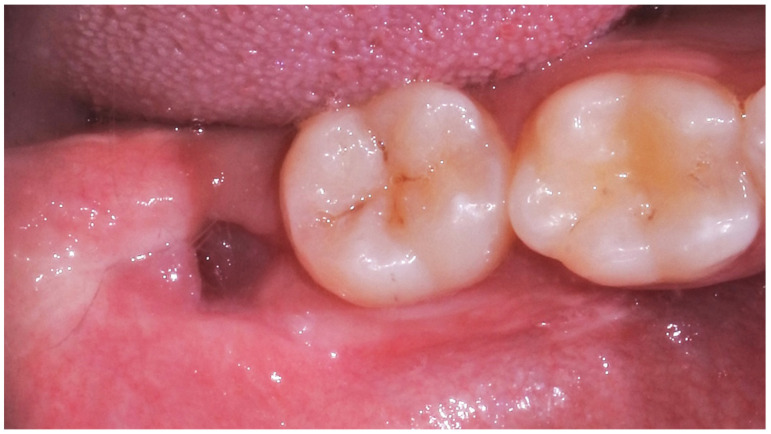
Post-surgical site (4.8) evaluation at 21-day follow-up.

Alveolitis, dehiscence and suppuration were also assessed through a detailed inspection of the post-surgical site, evaluating the presence of osteitis and/or purulent exudate (Figure 4).

Furthermore, the presence of neuralgic injury was evaluated using the light touch test, which consists of tracking the course of the inferior alveolar nerve by exercising a light pressure (“light touch”) at several points with a cotton swab. The patient reports the feeling of pressure or not, and consequentially, the area of paresthesia can be mapped. If this complication occurred, the patient was monitored regularly in order to assess the pattern of recovery after 1 month, 3 months, and 6 months, according to the standardized assessments [30,31,32].

### 2.3. Clinical Procedure

Before oral surgery intervention, all patients received non-surgical periodontal therapy coupled with oral hygiene instructions and motivation. At 7 days, tooth extraction was performed following a standardized surgical approach.

After local anesthesia with mepivacaine 2%, access to the third molar was achieved from the buccal aspect, and bone was eventually removed with a tungsten carbide round bur on a straight handpiece under continuous irrigation with sterile saline solution. If necessary, sectioning of the crown and roots was performed with a diamond fissure bur. After reaching adequate dental mobility through the dislocation maneuver, the tooth avulsion was performed. Any periapical lesion was removed with the use of an alveolar spoon during the alveolar revision, waiting for the clot to form.

All sharp bony edges were smoothened. At last, a tension-free closure of the alveolar socket was realized with horizontal mattress sutures and single-interrupted sutures 3/0 silk. Then, an ice pack was applied to the patient’s face for 20 min.

Based on the results of a recent systematic review [22], all the patients underwent systemic antibiotic prophylaxis (amoxicillin 875 mg + clavulanic acid 125 mg every 12 h per os) starting from 2 days before up to 4 days after surgery. Furthermore, an anti-inflammatory therapy (ibuprofen 600 mg per os; one tablet after 2 h and another one 6 h after surgery) was prescribed.

Patients were instructed to rinse with 0.20% chlorhexidine-based mouthwash three times a day for 10 days (starting 24 h after surgery). Finally, a module was delivered on how to report the intensity of the pain accused (VAS scale), post-operative instructions, and reminders of the follow-ups. The sutures were removed during the 7-day follow-up, and the pain module (VAS scale) was withdrawn.

### 2.4. Data Collection

For each selected patient, the following data were recorded at baseline: gender, age, systemic diseases, the reason for dental extraction, inclusion type (partial or total), and surgical site (dx or sin). The characteristics of lower IMTMs (angulation, root anatomy, IAN relationship, and presence of pericoronaritis) were evaluated on CT scan and classified according to Pell and Gregory classification (Table 1). Furthermore, the duration of oral interventions was also recorded.

Interincisal distance and all measurements for the evaluation of edema (Tr-Al, TR-Pog, Tr-Exo, Tr-Che, Go-Pog, Go-Exo, Go-Al, and Go-Che) were recorded at baseline and during follow-up (3, 7, and 21 days).

Finally, other complications (alveolitis, dehiscence, suppuration, and neuralgic injury) were assessed during clinical controls through an oral inspection of the post-surgical site.

### 2.5. Statistical Analysis

The data analysis was performed using a commercially available statistical software (IBM SPSS Statistics v.25, IBM Inc., Armonk, NY, USA). Gender, systemic diseases, reason for surgery, and characteristics of IMTMs (inclusion type, surgical site, Pell and Gregory classification, angulation, root anatomy, IAN relationship, and the presence of pericoronaritis) were recorded as frequencies and percentages, while mean and standard deviations (SDs) were calculated for age (years), duration of oral surgery (minutes), trismus (millimeters), and edema (millimeters).

Pain was registered with a VAS scale, and all other post-operative complications (trismus, edema, alveolitis, dehiscence, neuralgic injury, and suppuration) were reported as frequencies and percentages. Furthermore, the presence of edema was assessed by changing at least one of the facial or gnathological measurements.

A correlation analysis was performed to identify potential associations between pre- (systemic diseases, Pell and Gregory classification, angulation, IAN relationship), peri- (surgical time), and post- (trismus, edema) operative factors and VAS scores during follow-up. When a statistically significant correlation was identified, a regression analysis was performed. A *p*-value < 0.05 was accepted to identify a statistically significant difference.

## 3. Results

A total of 25 participants were selected from October 2019 to October 2021 at the Department of Oral Surgery—University of Naples Federico II. The characteristics of patients at baseline are shown in Table 2. Most participants were female (21; 84%) with a mean age of 25 ± 3 years. Systemic diseases were represented by multiple sclerosis (8%) and fibromyalgia (4%), while 88% of patients were in healthy condition (ASA index ≤ 2). The reasons for oral surgery were dysodontiasis (15; 60%), severe tooth decay (3; 12%), orthodontics (4; 16%) and periodontal (3; 12%). The inclusion type was partial in 15 patients (60%) and total in 10 (40%). Moreover, the lower IMTM was the tooth 4.8 in 56% of the cases and 3.8 in 44%.

Table 3 reports the characteristics of lower IMTMs analysed on CT scans. The most frequent classes, according to Pell and Gregory classification, were: BII (9; 36%), AII (7; 28%), BIII (5; 20%) and CII (4; 16%). The tooth angulations were vertical in 40% of the cases, mesio-inclined in 28%, and horizontal in 32%.

Radicular anatomy was represented in 52% of patients as fused, 44% as separated, and 4% as buttoned. The relationship with the inferior alveolar nerve (IAN) was, respectively, 56% of proximity >2 mm, 32% of proximity ≤2 mm, and 12% of continuity.

Pericoronaritis was recorded in 15 patients (60%) before oral surgery. At last, the average duration of intervention was 35 ± 5 min.

Post-operative pain was recorded using the Visual Analogic Scale, as reported in Table 4. The VAS values showed that most patients had mild pain (VAS score: 2–4) at 6 h (44%), 12 h (48%), 24 h (68%), and 48 (68%) after surgery. Pain and discomfort were reported in only 6 cases (24%; VAS score 2–4) at 72 h, while a VAS score of 0–1 was recorded in all patients after 7 days.

Table 5 shows the average interincisal distance differences from the baseline. Trismus was observed in 5 patients (20%) at 3-day follow-up, with an average reduction of mouth opening (interincisal distance) of 7 mm ± 3.

This reduction was 3 mm ± 3 at 7-day follow-up but only in 4 patients (16%), while no reduction of the physiological range of mouth opening (40–55 mm) was registered at 21 days.

Facial and gnathological measurements for the evaluation of edema are described in Table 6. The majority of patients (64%) showed edema at 3-day follow-up, with an increase in all parameters. However, these values decreased at 7-day follow-up as well as the number of patients with swelling (12%). All facial and gnathological measurements returned at baseline values at 21 days.

Table 7 reports other post-operative complications observed during the clinical inspection of the surgical site. While dehiscence and suppuration were not recorded, alveolitis was instead present in 12% of patients at 3-day follow-up, in 4% at 7 days, and in no case at 21 days.

Neuralgic injury represented by temporary paraesthesia was reported in one case (4%) at 7-day follow-up, where a buttoned root was in a continuity relationship with IAN. The area of paraesthesia was mapped by a light touch test using a cotton swab. The patient was monitored regularly at the Department of Oral Surgery—University of Naples Federico II, and spontaneous healing was observed after 3 months.

A linear regression analysis between VAS scores and patient- and tooth-related factors was performed, showing a statistically significant association (*p* < 0.05) between the duration of oral surgery and VAS scores at 6 (Table 8) and 12 h (Table 9).

Moreover, the logistic binary regression analysis identified systemic disease, Pell and Gregory classification, duration of oral surgery, VAS at 6 and 12 h, and trismus and edema at 3 and 7 days as predictive factors of post-operative complications (Table 10).

## 4. Discussion

The aim of this study was to evaluate the onset of complications following lower impacted third molar (IMTM) extraction. More specifically, it was sought to assess the patient’s perception related to post-operative pain and to identify potential risk predictors.

In this study, the majority of patients were female and needed IMTM extraction due to dysodontiasis. These data are confirmed by Kruger et al. [33], who reported that lower third molar inclusion more frequently affects the female gender.

Furthermore, Juodzbalys and Daugela [6] stated in their review that dysodontiasis is one of the most frequent causes of extraction, followed by carious lesions, resorption of adjacent teeth, pericoronaritis, periapical abscess, and the presence of a cyst or neoplasm.

Regarding the variables related to inclusion type, this study adopted Pell and Gregory classification, showing a prevalence of type BII (9; 36%), followed by AII (7; 28%), BIII (5; 20%), and CII (4; 16%). These patterns are in line with Santos et al. [34], who reported BII as the most frequent class of inclusion (26.4%) [34]. Moreover, class BII is the second most common position associated with secondary caries, preceded by class AII.

Position CIII was not reported in our study, although it is the most difficult position in terms of oral surgery procedure, as well as the most common class related to early and post-operative complications [34].

In this study, the majority of the third molars were located in vertical angulation (10; 40%). In contrast, Santos et al. [34] highlighted a majority of mesio-angular positions (41.8%) on a total of 1055 third lower molars analysed [34].

The IAN relationship was 56% of proximity > 2 mm, 32% of proximity ≤ 2 mm, and 12% of continuity.

However, there are other radiological classifications in order to evaluate the distance from the mandibular canal, as reported by the study of W.P. Smith [35] et al., in which on a total of 1589 teeth, 466 (29%) showed a distant relationship, 869 (55%) were close to the canal, and only 254 (16%) were deemed to be intimate to the canal [35]. Despite different evaluations, these results are partially in line with our study.

Pericoronaritis is a pathological condition due to the inflammation of the pericoronary follicle, reported in this study in 15 patients (60%). This inflammatory process is widely reported in the literature, and it is considered the most common reason for pain and discomfort. Smith et al. [35] stated the presence of pericoronaritis in 772 third molars (49%) that required dental avulsion [35].

A linear regression analysis was performed to identify potential associations between patient- and tooth-related factors and VAS scale. The results showed a positive correlation only for the duration of intervention with VAS at 6 and 12 h post-intervention. No correlations were found in subsequent follow up (24 h, 48 h, 72 h, and 7 days).

These data are in line with several other studies, which have demonstrated how the length of the surgical intervention is strictly related to the onset of early pain, as well as being a possible predictive factor of a longer recovery when the time of surgery is over 30 min [36,37]. Furthermore, Alvira-González et al. [38] reported that surgical time could also be considered a predictive variable for determining extraction difficulty [38,39].

A logistic binary regression analysis was also performed in order to evaluate potential predictive factors related to IMTM complications. The analysis showed a positive correlation for the following variables: systemic disease, Pell and Gregory classification, duration of oral surgery, VAS at 6 and 12 h, and trismus and edema at 3 and 7 days.

Many authors showed a correlation between post-operative complications and systemic diseases, not only with fibromyalgia but also with multiple sclerosis [40]. These pathological conditions indeed determine a systemic inflammatory state, with a massive release of IL-6 and a leukocyte activity deficit mainly detected from 2 to 6 h post-intervention. All these phenomena lead to a delay in the healing process and an increased risk of complications [27].

The position of IMTM, evaluated with Pell and Gregory classification, was shown to be, in this study, a predictive factor of post-operative discomfort. According to Bui et al. [8], mesio-angular impacted teeth were associated with a higher risk for post-operative complications. Moreover, Yuasa et al. [41] found that depth and ramus relationship/space available were associated with a more difficult extraction and, consequently, pain and discomfort.

Duration of oral surgery was also a predictive factor of pain, trismus, and edema. According to Alkadi et al. [16], the greater the operative time, the greater the probability of post-operative complications.

The assessment of trismus, measured by comparing the interincisal distance difference from baseline, showed a percentage of 20% at 3 days and 16% at 7 days, while there was a complete recovery by the 21st day of follow-up. These results are in accordance with De Menezes et al. [42], although it is very probable that these values have been achieved due to the use of non-steroidal anti-inflammatory drugs (NSAIDs). In our study, NSAIDs were administrated to all patients (ibuprofen 600 mg per os; one tablet after 2 h and another one 6 h after surgery), so due to the lack of a control group, we were not able to assess any statistical correlations.

With regard to swelling values, the results of this study showed the presence of edema in 64% of patients at 3 days after surgery, values that are significantly reduced at 7-day follow-up (12%). The swelling can be explained by the inflammatory and edema responses that occur as a result of surgical trauma.

This mechanism occurs mainly through the production of prostaglandins and cyclooxygenases, which are synthesized following arachidonic acid release from the cell membrane of cells at the surgical site [43]. In contrast, Yuasa et al. [41] reported high rates of edema from the third day after surgery to the seventh day, with a gradual onset and a peak at 48-h post-intervention.

Nevertheless, in this study, alveolar osteitis (AO) did not prove to be statistically significant, although it is one of the most common complications related to impacted third molars extraction. Therefore, Bartuli et al. [44] reported an incidence of AO between 4.1% to 32.6%, relating this to mild and severe pain.

Suppuration and dehiscence were also not detected, although these complications are often reported in the literature. Generally, in these cases, the treatment involves antibiotic therapy (clindamycin or amoxicillin) or a re-opening of the surgical site and curettage in case of refractoriness to therapy [45]. Rahpeyma et al. [46] highlighted how wound dehiscence could be avoided with a more conservative flap; nevertheless, this depends on the clinical case.

In this study, inferior alveolar nerve (IAN) continuity did not show to have any correlation with post-operative complications, although Albuquerque et al. [23] reported a positive association with a greater probability of nerve injury.

In our study, only one patient showed temporary paresthesia, which healed spontaneously after 3 months. Many studies in the literature report that paresthesia, mainly due to post-operative edema and swelling, generally tends to heal after 2–6 months [47]. Possible therapies to facilitate recovery after nerve injury include the administration of neurotrophic factors such as B12 vitamin, mecobalamin, or a stellate ganglion block, as suggested by Nogami et al. [47], despite being an invasive procedure.

The main objective of IMTM surgery is avulsion with minimal intra- and post-operative complications, performing the most conservative technique possible and supporting the patient in the post-intervention period.

There is much evidence of a relationship between surgical trauma and post-intervention complications [37]. Additionally, the size of the mucoperiosteum flap and the quantity of the osteotomy influenced the severity of pain and swelling [47]. Grossi et al. [48] demonstrated that raising a small flap without bone removal and/or tooth/root sectioning could cause patients to suffer more severe pain, presumably because of the excessive soft tissue damage and delayed wound healing [49]. Furthermore, Lo Giudice et al. [50] highlighted how an ostectomy performed with an ultrasonic tip showed the best results, preserving the bone morphology in both quantitative and qualitative analyses [50].

Therefore, a meticulous surgical planification with a CT exam is essential in order to minimize complications, especially intra-operatively, and to perform a correct ostectomy and odontotomy [49].

## 5. Conclusions

Although the small number of patients, the use of a subjective pain assessment method (VAS scale), and the prescription of an anti-inflammatory therapy represent limitations, the outcomes of this prospective cohort study suggest that the onset of pain and other post-operative complications increase in proportion to the duration of the surgical procedure, in accordance with the literature. Hence, an accurate evaluation of risk predictors and a conservative surgical technique will minimize complications or at least reduce recovery times and patient symptoms. Further investigations with a large sample and a more objective pain assessment method are needed to elucidate the best preventive and surgical protocols for the management of the complications related to third molar avulsion.

## Figures and Tables

**Table 1:** Pell and Gregory classification of lower impacted third molars (1933).

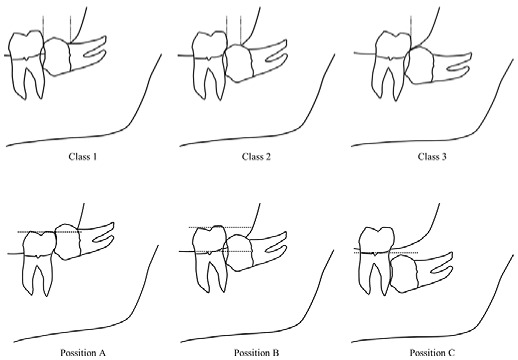	**Class**	
	Class I	There is enough space between the ramus and the distal surface of the second molar for the accommodation of the mesiodistal size of the crown of the third molar
	Class II	The space between the ramus and the distal surface of the second molar is less than the mesiodistal size of the crown of the third molar
	Class III	All or most of the third molar is located within the ramus
	**Position**	
	Position A	The highest point of the tooth is on level or above the occlusal plane of the second molar
	Position B	The highest point of the tooth is below the occlusal plane but above the cervical line of the second molar
	Position C	The highest point of the tooth is below the cervical line of the second molar

**Table 2:** Characteristics of selected patients at baseline.

	Patients (N = 25)		%
Gender	Female = 21 Male = 4		84% 16%
Mean age	25 ± 3 years		
Systemic disease	Healthy (ASA Index ≤ 2) = 22 Multiple sclerosis (MS) = 2 Fibromyalgia (FMS) = 1		88% 8% 4%
Reason of surgery	Dysodontiasis Severe tooth decay Orthodontics Periodontal	=15 =3 =4 =3	60% 12% 16% 12%
Inclusion type	Partially impacted Totally impacted	=15 =10	60% 40%
Surgical site	Third molar dx (4.8) Third molar sin (3.8)	=14 = 11	56% 44%

**Table 3:** Characteristics of lower impacted third molars (IMTMs).

	Patients (N = 25)		%
Classification (Pell and Gregory)	BII AII BIII CII	=9 =7 =5 =4	36% 28% 20% 16%
Angulation	Vertical Mesio-inclined Horizontal	=10 =7 =8	40% 28% 32%
Root anatomy	Fused roots Separated roots Buttoned roots	=13 =11 =1	52% 44% 4%
IAN relationship	Proximity > 2 mm Proximity ≤ 2 mm Continuity	=14 =8 =3	56% 32% 12%
Pericoronaritis	Yes No	=15 =10	60% 40%
Average duration of oral surgery	35 ± 5 min		

**Table 4:** VAS Scores.

Patients (N = 25)						
VAS Score	6 h	12 h	24 h	48 h	72 h	7 Days
0–1	4 (16%)	4 (16%)	4 (16%)	4 (16%)	19 (76%)	25 (100%)
2–4	11 (44%)	12 (48%)	17 (68%)	17 (68%)	6 (24%)	0 (0%)
5–7	8 (32%)	8 (32%)	4 (16%)	4 (16%)	0 (0%)	0 (0%)
8–9	2 (8%)	1 (4%)	0 (0%)	0 (0%)	0 (0%)	0 (0%)
10	0 (0%)	0 (0%)	0 (0%)	0 (0%)	0 (0%)	0 (0%)

**Table 5:** Average interincisal distance difference from baseline.

Patients (N = 25)			
Follow Up	Trismus		Average Interincisal Distance Difference (from Baseline)
3-day	Yes No	=5 (20%) =20 (80%)	−7 mm ± 3
7-day	Yes No	=4 (16%) =21 (84%)	−3 mm ± 3
21-day	Yes No	=0 (0%) =25 (100%)	0 mm ± 3

**Table 6:** Average facial and gnathological measurements differences from baseline.

Patients (N = 25)			
Follow Up	Edema	Facial Measurements	Average Facial and Gnathological Measurements Differences (from Baseline)
3-day	Yes = 16 (64%) No = 9 (36%)	Trago-nasal border (Tr-Al)	+3.8 mm ± 1.5
		Trago-anatomic pogonion (TR-Pog)	+3.8 mm ± 1.5
		Trago-eye’s external corner (Tr-Exo)	+3.8 mm ± 1.4
		Trago-labial commissure (Tr-Che)	+3.7 mm ± 1.5
		Anatomic Gonion-anatomic Pogonion (Go-Pog)	+3.8 mm ± 1.5
		Anatomic Gonion-eye’s external corner (Go-Exo)	+3.8 mm ± 1.4
		Anatomic Gonion-nasal border (Go-Al)	+3.8 mm ± 1.6
		Anatomic Gonion-labial commisure (Go-Che)	+3.7 mm ± 1.4

7-day	Yes = 3 (12%) No = 22 (88%)	Trago-nasal border (Tr-Al)	+2.3 mm ± 1
		Trago-anatomic pogonion (TR-Pog)	+2.4 mm ± 1
		Trago-eye’s external corner (Tr-Exo)	+2.3 mm ± 1
		Trago-labial commissure (Tr-Che)	+2.3 mm ± 1
		Anatomic Gonion-anatomic Pogonion (Go-Pog)	+2.4 mm ± 1
		Anatomic Gonion-eye’s external corner (Go-Exo)	+2.3 mm ± 1
		Anatomic Gonion-nasal border (Go-Al)	+2.3 mm ± 1
		Anatomic Gonion-labial commisure (Go-Che)	+2.3 mm ± 1

21-day	Yes = 0 (0%) No = 25 (100%)	/	/

**Table 7:** Other post-operative complications.

Follow up	Complication	Patients (N = 25)	%
3-day	Alveolitis	Yes = 3 No = 22	12% 88%
	Dehiscence	Yes = 0 No = 25	0% 100%
	Nevralgic injury	Yes = 1 No = 24	4% 96%
	Suppuration	Yes = 0 No = 25	0% 100%

7-day	Alveolitis	Yes = 1 No = 24	4% 96%
	Dehiscence	Yes = 0 No = 25	0% 100%
	Nevralgic injury	Yes = 1 No = 24	4% 96%
	Suppuration	Yes = 0 No = 25	0% 100%

21-day	Alveolitis	Yes = 0 No = 25	0% 100%
	Dehiscence	Yes = 0 No = 25	0% 100%
	Nevralgic injury	Yes = 1 No = 24	4% 96%
	Suppuration	Yes = 0 No = 25	0% 100%

**Table 8:** Correlation between VAS scores at 6 h and patient- and tooth-related factors.

**Model Summary**						
Model	R	R-square	Adapted R-square	Std. error of the estimate		
1	0.828 ^a^	0.685	0.496	0.605		
**ANOVA**						
1		Sum of squares	df	Mean Square	F	Sign.
	Regression	11.946	9	1.327	3.624	0.14 ^a^
	Residual	5.494	15	0.366		
	Total	17.440	24			
**Coefficients**						
1		Non-standardized coefficients		Standardized coefficients		
		B	Standard Error	Beta	t	Sign.
	(Constant)	−3.384	2.127		−1.591	0.133
	Pell and Gregory classification	0.255	0.299	0.243	0.854	0.406
	IAN relationship	0.392	0.280	0.327	1.401	0.182
	Duration of oral surgery	1.279	0.384	0.808	3.329	0.005
	Systemic disease	0.405	0.428	0.284	0.946	0.359
	Edema at 3-day	−0.038	0.037	−0.240	−1.053	0.309
	Edema at 7-day	−0.063	0.089	−0.196	−0.709	0.489
	Trismus at 3-day	0.550	0.385	0.319	1.428	0.174
	Trismus at 7-day	−0.128	0.689	−0.061	−0.186	0.855
	Angulation	0.107	0.204	0.108	0.525	0.607

^a^ Predictors: (costant) Pell and Gregory classification, IAN relationship, duration of oral surgery, systemic diseases, edema at 3- and 7- day, trismus at 3- and 7- day, angulation.

**Table 9:** Correlation between VAS scores at 12 h and patient- and tooth-related factors.

**Model Summary**						
Model	R	R-square	Adapted R-square	Std. error of the estimate		
1	0.839^a^	0.703	0.526	0.537		
**ANOVA**						
1		Sum of squares	df	Mean Square	F	Sign.
	Regression	10.242	9	1.138	3.954	0.009 ^a^
	Residual	4.138	15	0.288		
	Total	14.560	24			
**Coefficients**						
1		Non-standardized coefficients		Standardized coefficients		
		B	Standard Error	Beta	t	Sign.
	(Constant)	−3.971	1.886		−2.106	0.052
	Pell and Gregory classification	0.119	0.265	0.124	0.448	0.660
	IAN relationship	0.234	0.248	0.214	0.944	0.360
	Duration of oral surgery	1.209	0.341	0.836	3.351	0.003
	Systemic disease	−0.706	0.380	−0.541	1.858	0.083
	Edema at 3-day	−0.017	0.032	−0.118	−0.536	0.600
	Edema at 7-day	0.034	0.079	0.115	0.429	0.674
	Trismus at 3-day	0.458	0.342	0.290	1.340	0.200
	Trismus at 7-day	0.582	0.611	0.305	0.952	0.356
	Angulation	0.117	0.181	0.129	0.646	0.528

^a^ Predictors: (costant) Pell and Gregory classification, IAN relationship, duration of oral surgery, systemic diseases, edema at 3- and 7- day, trismus at 3- and 7- day, angulation.

**Table 10:** Logistic binary regression analysis.

**Classification Table ^a,b^**									
Observed			Predicted						
			Complications		Percentage correct				
			No (0)	Yes (1)					
Step 0	Complications	No (0)	21	0	100.0				
		Yes (1)	4	0	0.0				
	Overall percentage				84.0				
**Variables in the Equation**									
Step 0		B	S.E.	Wald	df	Sign.		Exp (B)	
	Constant	−1.658	0.546	9.239	1	0.002		0.190	
**Variables not in the Equation ^a^**									
Step 0			Score		df		Sign.		
	Variables	Angulation	0.043		1		0.836		
		Pell and Gregory classification	6.361		1		0.12		
		IAN relationship	1.895		1		1.169		
		Duration of oral surgery	8.622		1		0.003		
		Systemic disease	14.187		1		0.000		
		VAS at 6-h	9.505		1		0.002		
		VAS at 12-h	4.723		1		0.030		
		VAS at 24-h	3.720		1		0.054		
		VAS at 48-h	3.720		1		0.054		
		VAS at 72-h	1.765		1		0.184		
		Trismus at 3-day	6.857		1		0.009		
		Trismus at 7-day	9.003		1		0.003		
		Edema at 3-day	11.049		1		0.001		
		Edema at 7-day	6.512		1		0.011		

^a^ Constant is included in the model. ^b^ The cut value is 0.500.

## Data Availability

All data are available from the corresponding author upon reasonable request. The data are not publicly available due to privacy restrictions.
